# A gene profiling deconvolution approach to estimating immune cell composition from complex tissues

**DOI:** 10.1186/s12859-018-2069-6

**Published:** 2018-05-08

**Authors:** Shu-Hwa Chen, Wen-Yu Kuo, Sheng-Yao Su, Wei-Chun Chung, Jen-Ming Ho, Henry Horng-Shing Lu, Chung-Yen Lin

**Affiliations:** 1 Institute of Information Science, Academia Sinica, 128 Academia Road, Section 2, Nankang, Taipei, 115 Taiwan; 2 Institute of Statistics, National Chiao Tung University, Assembly Building I, 1001 Ta Hsueh Road, Hsinchu, 30010 Taiwan; 3 Bioinformatics Program, Taiwan International Graduate Program, Institute of Information Science, Academia Sinica, 128 Academia Road, Section 2, Nankang, Taipei, 115 Taiwan; 4 Institute of Biomedical Informatics, National Yang-Ming University, No. 155, Sec. 2, Linong St., Beitou District, Taipei City, 112 Taiwan; 5 Division of Biostatistics and Bioinformatics, Institute of Population Health Sciences, National Health Research Institutes, 35 Keyan Road, Zhunan, Miaoli County, 35053 Taiwan; 6 Institute of Fisheries Science, College of Life Science, National Taiwan University, No. 1, Sec. 4, Roosevelt Rd, Taipei, 10617 Taiwan

## Abstract

**Background:**

A new emerged cancer treatment utilizes intrinsic immune surveillance mechanism that is silenced by those malicious cells. Hence, studies of tumor infiltrating lymphocyte populations (TILs) are key to the success of advanced treatments. In addition to laboratory methods such as immunohistochemistry and flow cytometry, in silico gene expression deconvolution methods are available for analyses of relative proportions of immune cell types.

**Results:**

Herein, we used microarray data from the public domain to profile gene expression pattern of twenty-two immune cell types. Initially, outliers were detected based on the consistency of gene profiling clustering results and the original cell phenotype notation. Subsequently, we filtered out genes that are expressed in non-hematopoietic normal tissues and cancer cells. For every pair of immune cell types, we ran t-tests for each gene, and defined differentially expressed genes (DEGs) from this comparison. Equal numbers of DEGs were then collected as candidate lists and numbers of conditions and minimal values for building signature matrixes were calculated. Finally, we used v -Support Vector Regression to construct a deconvolution model. The performance of our system was finally evaluated using blood biopsies from 20 adults, in which 9 immune cell types were identified using flow cytometry. The present computations performed better than current state-of-the-art deconvolution methods.

**Conclusions:**

Finally, we implemented the proposed method into R and tested extensibility and usability on Windows, MacOS, and Linux operating systems. The method, MySort, is wrapped as the Galaxy platform pluggable tool and usage details are available at https://testtoolshed.g2.bx.psu.edu/view/moneycat/mysort/e3afe097e80a.

## Background

Cancers comprise a group of diseases that are characterized by uncontrolled growth of abnormal immortalized cells that can spread to other parts of the body and finally deplete resources. Hanahan and Weinberg suggested biological capabilities and hallmarks of a multistep process toward the development of human tumors [1, 2]. Among these, tumors have strategies for evading immune destruction. In contrast, the immune system sometimes over-functions and incorrectly identifies normal cells, leading to convalescence and autoimmune disease. Hence, as regulators of immunity, various immune checkpoints achieve an equilibrium of system responses. Recently, cancer cells were shown to interact with immune checkpoints and inhibit T cell activation as an immune evasion strategy [3].

Immunotherapy is a novel strategy in which immune checkpoint components are manipulated, in particular leading to blockade of T cell activation. Interactions between immune cell receptors and ligands are essential targets of immune checkpoint blockade which involves many immune cell types and distinct pathways that are incompletely understood. Therefore, understanding the composition of immune cells in tissues is central to studies of immune checkpoints with the ultimate aim of developing immunotherapy. Flow cytometry and immunohistochemistry analyses have long been developed to define cell compositions but can’t be easily applied to resolve immune cell types from all kind of cancers. In contrast, whole transcriptome profiling methods such as microarray and next generation sequencing can be developed as in silico methods for defining compositions of a panel of cell types that are defined using mixed gene profiling of cell-type specific genes.

Gene expression deconvolution methods have been developed for several years and most methods regard deconvolution as a linear problem. Strictly speaking, the expression level of gene *i* in mixture *j* is the sum of its expression in *r* cell types, as indicated by the following equation:

1 $$ {M}_{ij}={\Sigma}_{k=1}^r{S}_{ik}{F}_{kj} $$

where *S*_*ik*_ is specific gene expression in cell type *k* and *F*_*kj*_ is the proportion of cell type *k* in mixture *j*. The general model can be expressed as an approximate matrix problem as follows: *M* ≈ *S* × *F*, and the problem can be solved from the view of an equation-solving question.

Abbas et al. [4] and Clarke et al. [5] were the first to propose deconvolution methods, and Gong et al. [6] and Zhong et al. [7] used quadratic programming as an optimization tool to solve the problem. In further studies, Gaujoux et al. [8] applied non-negative matrix factorization to deconvolution and Qiao et al. [9] and Liebner et al. [10] introduced statistics to solve the deconvolution problem. Finally, Newman et al. [11] developed the novel strategy CIBERSORT to solve the linear equation, and comparisons with the six deconvolution methods listed above by benchmarking on mixture expression data showed that the method agreed with flow cytometry data and outperformed other methods.

Tumor infiltrating lymphocytes (TILs) include various cell types in variable proportions, and leave the bloodstream to infiltrate into cancer tissues. TILs are associated with tumor growth, cancer progression, and patient prognoses [12]. Thus, assessments of the composition of TILs are fundamental for developing effective immunotherapies. In this study we analyzed the composition of TILs from mixed tissue gene profiling data and revised analyses for better performance.

## Methods

The deconvolution method in this study is mainly adjusted from the strategy of CIBERSORT. Table 1 describes the datasets of 22 immune cell types collected by Newman et al. [11] from 11 major leukocyte types. At least two replicates for each cell types are included in this study.

**Table 1:** A brief of the 113 microarrays used as the deconvolution dataset

Major leukocyte types	Subtypes	Number of replicates	
B cells	B cells naïve	7	*
	B cells memory	8	*
PCs	Plasma cells	7	
CD8 T cells	T cells CD8	4	*
CD4 T cells	T cells CD4 naïve	3	*
	T cells CD4 memory resting	3	*
	T cells CD4 memory activated	3	*
	T cells follicular helper	3	
	T cells regulatory (Tregs)	2	
Gamma delta T cells	T cells gamma delta	2	*
NK cells	NK cells resting	4	**
	NK cells activated	11	**
Monocytes and Macrophages	Monocytes	11	*
	Macrophages M0	12	
	Macrophages M1	3	
	Macrophages M2	3	
Dendritic cells	Dendritic cells resting	6	
	Dendritic cells activated	6	
Mast cells	Mast cells resting	2	
	Mast cells activated	2	
Eos	Eosinophils	2	
PMNs	Neutrophils	8	

Remarks: * and ** are cell types that used in the validation datasets (PBMC from 20 adults). Both activated and resting NK cells are counted as NK cells in the prediction on the benchmark experiment

### Implement resources

We develop the algorithms using R (version 3.1.1) and the following packages: preprocessCore (version 1.34.0), limma (version 3.28.31), geneplotter (version 1.50.0), qvalue (version 2.4.2), genefilter (version 1.54.2), plyr (version 1.8.4), and e1071 (version 1.6–7). The algorithms are then exam on Windows 10 and Ubuntu Linux 16.04 server. Note that the version of R and associated packages may be varied between operating systems.

To obtain usability and extensibility for a customized workflow, we adopt the proposed algorithms to a pluggable tool, MySort, and integrate the tool into the Galaxy platform.

### Research design

**Fig. 1:**
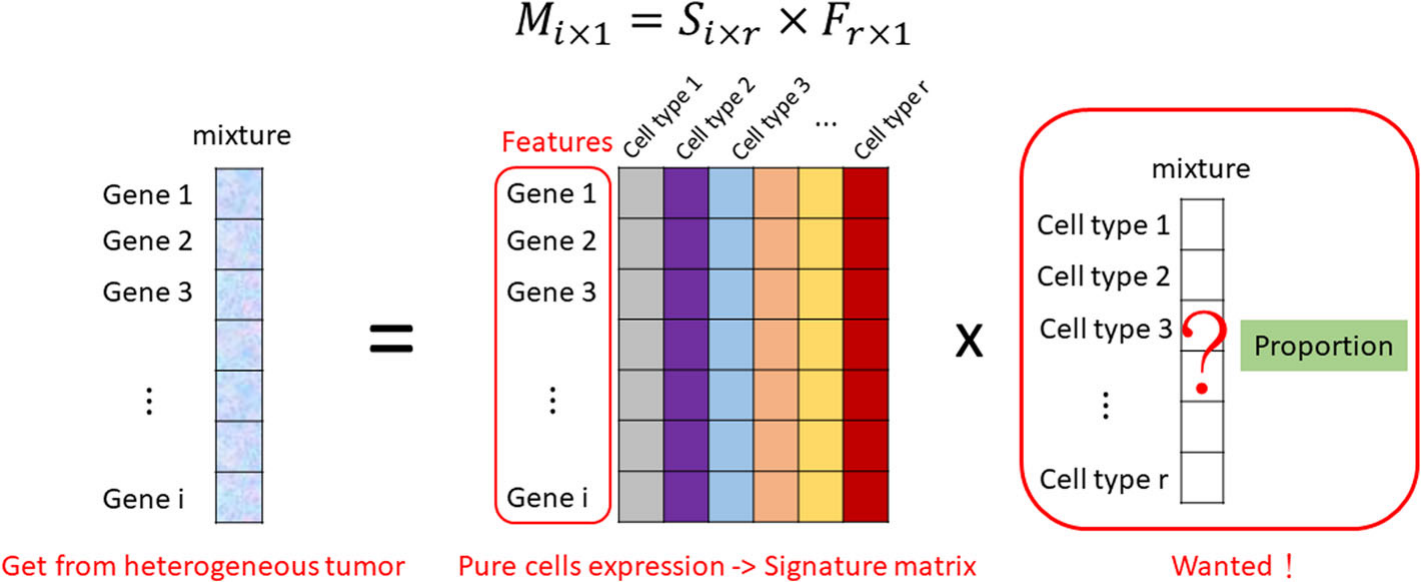
The deconvolution model

As mentioned previously, the deconvolution model can be presented as a matrix function. We denote the transcriptome expression level of a cell type consists of *i* genes. The transcription profile of a cancer biopsy will be the summation of each gene expression level cross the proportion of all cell types in the biopsy. Since we are concerning only in the composition of a particular panel of cell types, we pick *t* genes, a subset of genes that are only expressed in the given cell panel, to build a subset of gene expression matrix, namely signature matrix, *S*. Thus, *S* is a matrix with dimension of *t* rows and *r* columns where *r* represents the number of cell types in the given cell panel, the immune cells in this study. When *S* is defined, the vector *F*, the proportion of each cell type in the given panel, can be solved out. Hence, deconvolution can be divided into two parts: constructing a signature matrix and solving linear equation for *F* (Fig. 1).

### The signature matrix

**Fig. 2:**
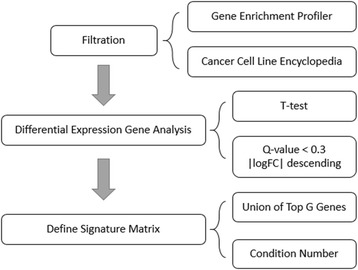
Steps of constructing signature matrix

The main strategy for choosing cell-type-specific genes is to do differential gene expression analysis. The following diagram explains the method of constructing signature matrix step by step (Fig. 2).

Data from the 22 cell types (the signature set, 113 arrays) are quantile normalized before detecting differentially expressed genes. In order to prevent the datasets containing bias experiment result, clustering on gene profiling is applied. The clustering method is complete linkage and in the Euclidean distance. The inconsistency of gene profiling clusters and cell type labels is further analyzed for an advanced outlier judgment. Correlation between arrays in the same cell type is calculated using Pearson correlation. For each array of a certain cell type, we judge the problematic cluster if any single array shows low gene expression correlations (R < 0.85) to the others in the same cell phenotype (i.e., more than 2/3 of the related array-to-array pairs within a cluster).

To define a set of feature genes for deconvolution, we first eliminate genes that are unsuitable for building model. Two methods were adopted. We use datasets and enrichment score (ES) described in Benita et al. [13] to define genes that expressed in normal tissues by the criteria of ES > 0 in more than 5% of observed tissue types. Besides, genes that expressed in cancer cells are collected from cancer cell line encyclopedia [14] in the criteria of log2 transformed expression level > 7. The two lists are used as black lists to remove genes expressed in normal tissues and cancer cells from the candidate list.

To select genes that can be the representative features of a cell type, we run the statistical analysis between each two immune cell type pairs. Firstly, the differentially expressed genes (DEGs) are detected using two-sided unequal variance T-test with a significant criterion in q-value < 0.3. Secondly, we sort the DEGs of each comparing pair by the absolute value of log fold change of gene expression level in descending order. Third, a top G (G = 5 to 100) ranked DEGs are selected from each pair to build a union set of a signature gene list (top G signature gene list) and to derive top G signature matrix, the expression profiles for each top G signature gene list. Condition number [15] which is associated with the linear equation is introduced to define the choice of G and calculated with “kappa” function in R.

### ν-support vector regression

First, gene expression profiles from both the signature set and the validation set (described in benchmark method) are quantile normalized. To avoid inconsistency of data range, a preprocessing procedure is introduced. We use a standardized score by converting the data to z score (mean = 0, variance = 1). Furthermore, genes in the signature matrix may be not always included in the mixture data set. We use the overlaps of genes as the final features for building the model.

The strategy to solve this regression problem is a machine learning method called *ν* − *support vector regression* (*ν*-SVR). In this study, support vectors represent a particular subset of genes in the signature matrix. *υ*-SVR is adopted in this study by “svm” function of R in package, “e1071”, with linear kernel. Furthermore, *F* have the best result with *υ* = {0.25,0.5,0.75}, where the evaluation method is to estimate the lowest root mean square error between M and S × F. Negative coefficients for regression solved by SVR are set to be 0, and then all coefficients are normalized to be summed to 1. Finally, the estimation of relative proportions for cell types is solved out, and correlations and root mean squares between M and S × F for each sample are evaluated.

### Benchmark method

The deconvolution method was benchmarked with flow cytometry results of twenty adult blood biopsies used in CIBERSORT study [11]. Since there are only nine cell types were labeled, we extract the prediction of these types and rescale the sum of the nine prediction value to 1. We use the cell type frequencies determined by flow cytometry as the standard and calculate correlation (Pearson correlation) and root mean square error (RMSE) of the derived expression level of signature genes to the true value to evaluate the performance of prediction.

## Results

### The agreement of cell phenotypes to gene expression profile clustering

**Fig. 3:**
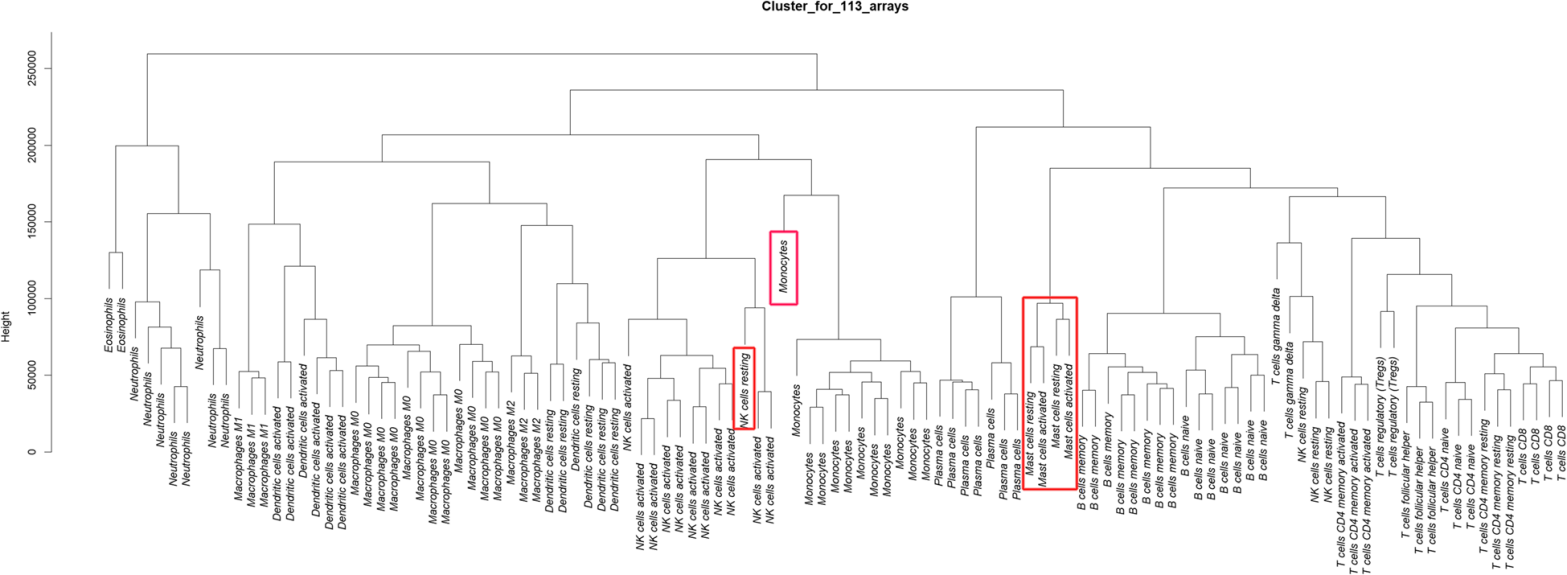
Hierarchical clustering for 113 arrays to detect the consistency between cell phenotypes and gene expression pattern. Arrays with problematic grouping are boxed in red

Initially, we retrieved data for building a deconvolution model. After a data preprocessing step, we clustered the deconvolution dataset (113 arrays) according to gene expression profiles. Although replicates of each cell type were expected to be clustered into the same group, one array of resting NK cells was inserted into the activated NK cells group and one array of monocytes is far from its group (Fig. 3), and arrays of resting and activated mast cells were arranged without clear segregation. Therefore, we performed Pearson correlations between arrays of cell types to identify outlying arrays. One array from monocytes and another from resting NK cells were excluded according to weaker correlations than those between others within the group. In addition, we decided to merge two cell types, resting and activated mast cells, into a single mast cell category due to the sparse of evidence on segregation.

### Construct signature matrix

**Fig. 4:**
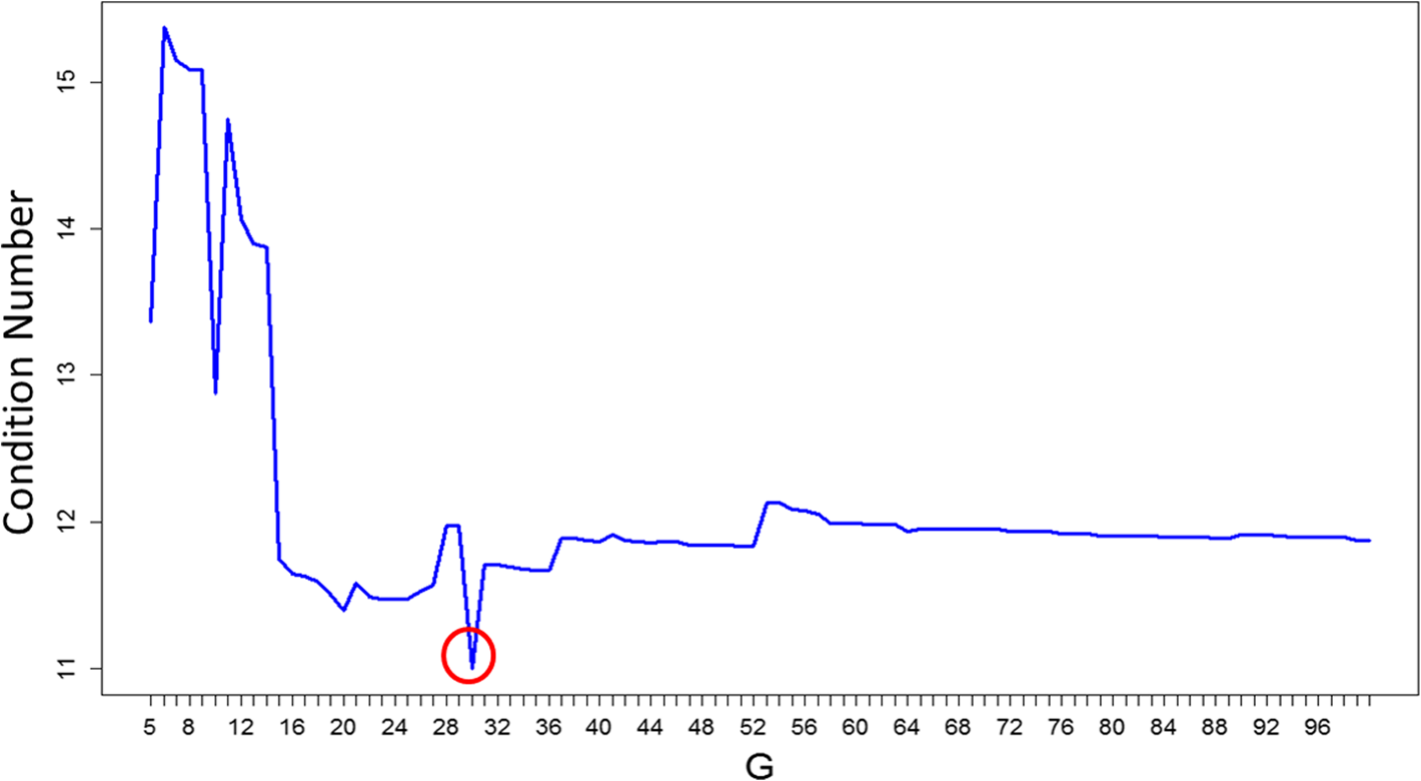
The condition number of iteration for G

After filtration processes, pairwise comparisons of cell types were performed using t-tests. The top G ranked DEGs (G = 5~ 100) were then joined to give the top G gene list for each G value. Subsequently, we calculated the numbers of conditions for each top G gene list and defined the final gene list to build the signature matrix as the list with the lowest number of conditions. The final results were G = 30, number of conditions = 10.99, and number of signature genes = 603 (Fig. 4).

**Fig. 5:**
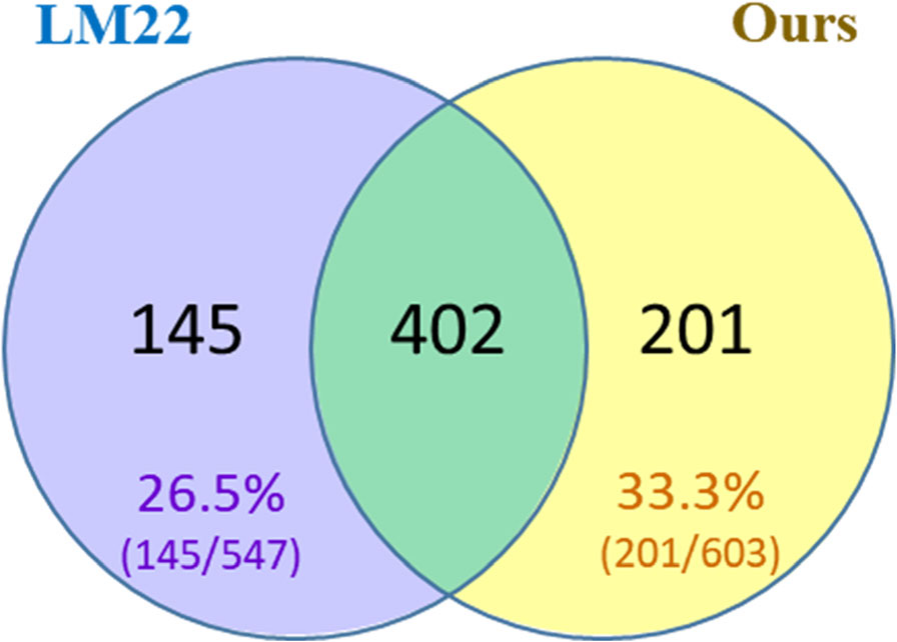
Venn-diagram for a number of genes of two signature gene sets. (LM22 is signature matrix of CIBERSORT)

The top G gene list (G = 30) was then compared to the LM22 as defined using CIBERSORT. In the Venn-diagram for numbers of genes in Fig. 5, two-thirds of our signature genes overlapped with LM22, although 201 genes were uniquely defined by us and 145 were uniquely defined by CIBERSORT.

### Benchmark method

**Fig. 6:**
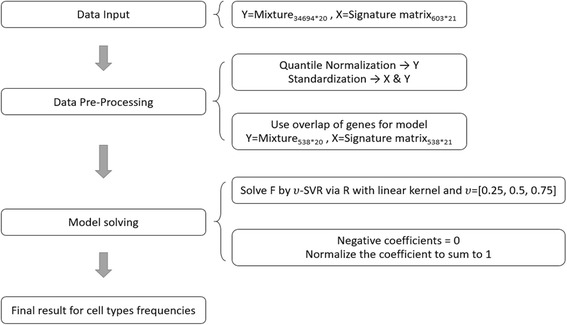
Workflow of the deconvolution method

We used cell composition data from reported peripheral blood mononuclear cells as a validation to benchmark the performance of deconvolution methods, and as mentioned in the methods, nine cell types were resolved by flow cytometry in the validation dataset. We then recalculated relative proportions of all cell types, but recalculated the relative portion of nine relevant types to a sum of 1. Similarly, neither mast cells nor subtyped resting and activated mast cells from CIBERSORT were typed in the validation dataset. The process is illustrated in Fig. 6.

We compared our prediction with the result provided in CIBERSORT and estimated performance according to RMSE (Table 2) and Pearson correlations (Table 3) for each cell type. These comparisons indicate that the present deconvolution method outperforms CIBERSORT. The differences between our approach and CIBERSORT are summarized in Table 4.

**Table 2:**
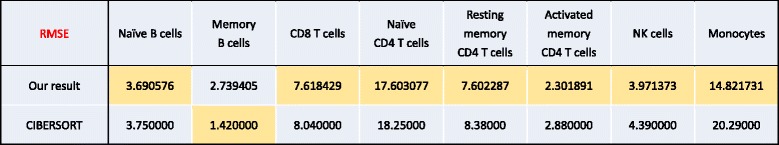
The performance of our deconvolution method and in CIBERSORT, evaluated by RMSE of signature genes

Method in better performance (lower RMSE) is marked yellow

**Table 3:**
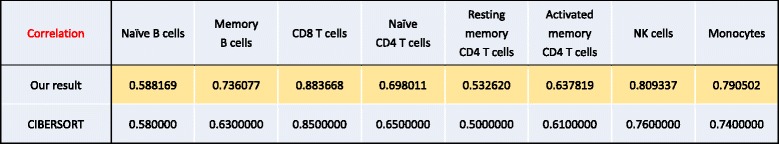
The performance of our deconvolution method and in CIBERSORT, evaluated by Pearson correlation of cell proportion

Method in better performance (higher correlation) is marked yellow

**Table 4:**
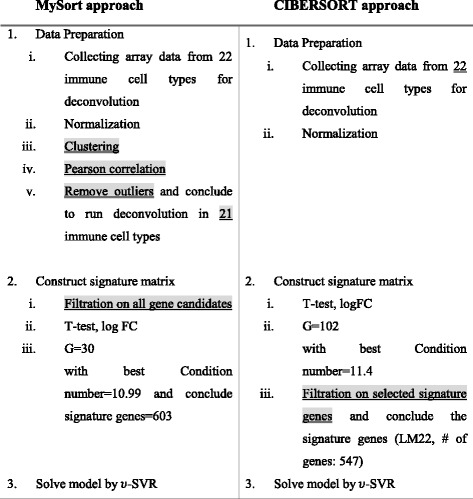
Comparison the difference of our approach with CIBERSORT. Major differences are marked gray and underlined

Major differences are marked gray and underlined

### Galaxy plugin

**Fig. 7:**
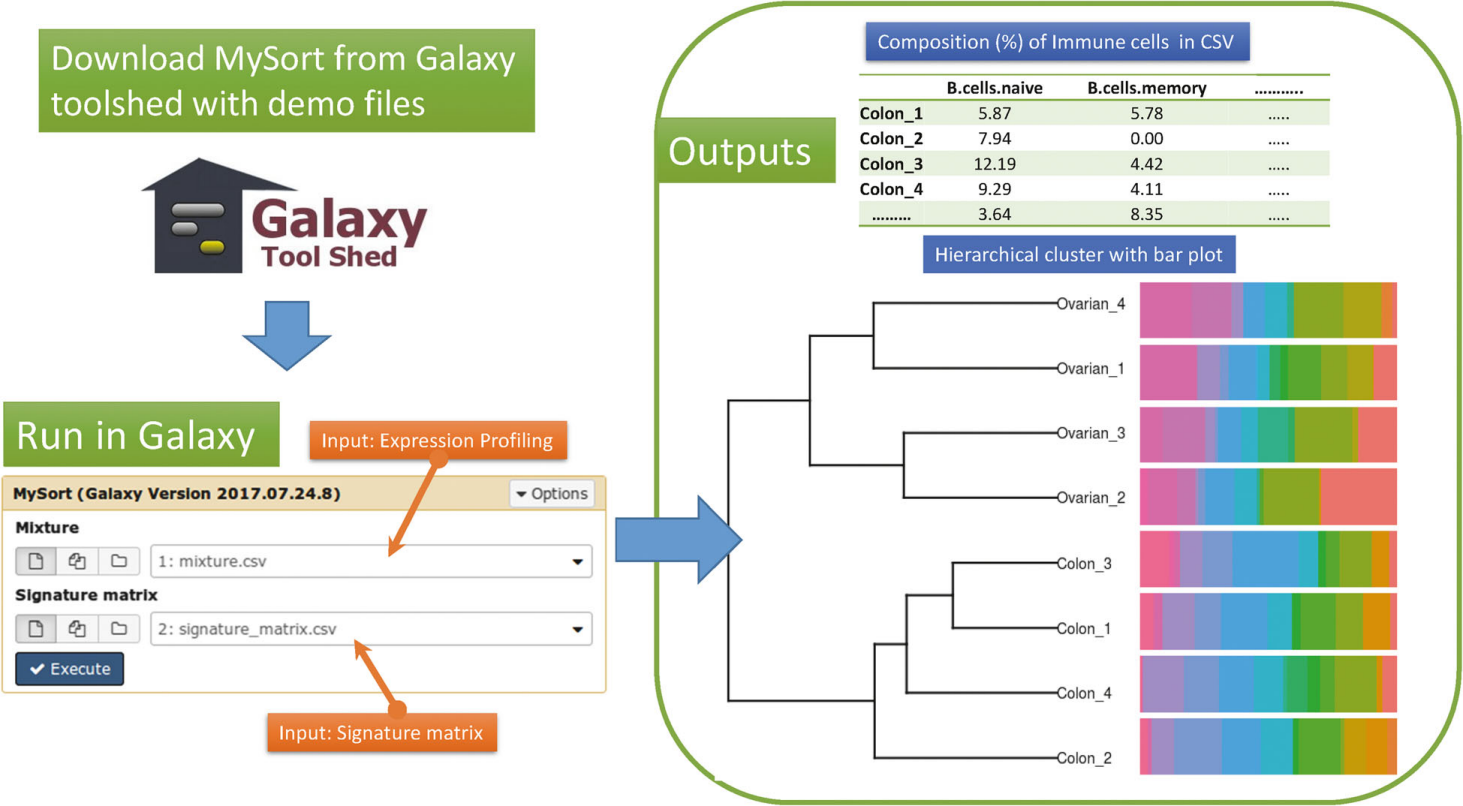
The implementation of galaxy plugin for the deconvolution method

Our validation experiments show that the present workflow outperforms previous models for most cell types. To improve access to the research community interesting in detecting infiltrating immune cells, we have implemented the present algorithms MySort in R and wrapped as a Galaxy platform pluggable tool and provide usage details in the Galaxy toolshed https://testtoolshed.g2.bx.psu.edu/ view/moneycat/mysort/e3afe097e80a (Fig. 7). Using mixture expression profiling data (uploaded by the user, indexed in gene symbols with samples arranged by column) and the signature matrix (provided by this study) as inputs, MySort generates the immune cell compositions for each expression profile in csv format and a combined diagram to present the resolved cell proportion in a bar chart plot and a hierarchical clustering plot for relatedness among submitted samples.

## Discussions

Signature genes play important roles in gene expression deconvolution computations. Additionally, outlier detection, gene list filtration, and support vector regression were central to the positive outcomes of our model. We also revised the deconvolution process and discovered issues that were not properly dealt with previously, including uncertain accuracy of representative data matrixes for each immune cell type and unequal contributions of DEG pairs that are used to build signature matrixes.

Removing the outliers is an important preliminary step for any in silico model. We revised the cell phenotype to the profiling clustering result and identified possible outliers and improper typing groups. Subsequently, we excluded two arrays and merged two classes in the signature set. In addition, we identified limitations of data replicates in some cell types as a cause of decreased confidence in DEGs with weak statistical power. Further technical difficulties, such as inconsistency of cell type definitions in different laboratories may introduce additional problems for data analysis. For example, gamma delta T cells are apparently difficult to identify with certainty, and as stated in the CIBERSORT study, few microarrays of gamma delta T cells contain more than two replicates.

We assumed that the present deconvolution model is linear. Thus, to realize and simplify the model, we selected features as genes that are specifically expressed in certain cell types. In addition, we used support vector regression (SVR) to resolve the regression problem of cell compositions. SVR was developed from a support vector machine as an optimization approach for binary classification problems. This method defines hyperplanes that separate classes with the largest possible margin by maximizing the distance from the hyperplane to the nearest data point. In contrast, SVR seeks a hyperplane that fits the data points in a tube of width *2ε*. Hence, in ε-based SVR (ε-SVR), data points at most ε values deviate from the hyperplane. Consequently, ε-SVR does not focus on data points with the ε-tube, points out of the tube are support vectors (SVs), and distances from SV’s to the boundary of the tube are evaluated by the loss function. Similar to *ε*-SVR,*υ*-SVR uses *υ* to provide a more convenient control over the number of SVs and training errors. However, the required SV chosen by the SVR model are some important genes are selected for solving the regression function. These properties of SVR are key to the enhanced performance of the present model in comparison with other traditional methods for solving regression problems.

Gene list filtration directly introduces a black list of unrelated genes that are either expressed in cell types other than those of interest or may interfere with the deconvolution strategy. Although this is an effective strategy for building deconvolution models, the timing of filtration can alter the selection of signature genes greatly. Thus, we used the filtration step before selecting top G ranked DEGs, whereas CIBERSORT filtered genes in the black list after selecting top G ranked DEGs. Consequently, our model achieved G = 30 with 10.99 best conditions and 603 union gene sets, whereas CIBERSORT achieved G = 102 with 11.4 best conditions and 547 union gene sets. Moreover, the processes in CIBERSORT lead to the use of unequal numbers of genes to distinguish cell types.

## Conclusions

Gene expression deconvolution methods can be used to reveal defined cell types from transcriptomes of samples with mixed cell types, and are demonstrably powerful strategies for identifying TILs in cancer tissues through reanalyzes of accumulated microarray databanks. Due to the heavy task of reanalysis, we implemented the algorithm, improved its performance, and then packed it as a portable application.

The present version in MySort regards the deconvolution model as a linear model. However, nonlinear methods may be applied to gene expression deconvolution, and machine learning has recently been shown to have good predictive performance. Furthermore, deep learning is an advanced method for nonlinear problems. Hence, further studies are required to develop machine learning and deep learning methods to decipher large databases, and to train the model using the data to make good predictions in gene expression deconvolution.

Finally, new high-throughput technologies such as next generation sequencing and single cell technologies are considered advanced techniques for gene profiling. Theoretically, all cell components could be resolved with knowledge of expression profiles of all cell types. Moreover, greater numbers of profiled cell types will necessitate strategies for classifying them. Subsequently, resolving gene profiling at the single cell level will drive deconvolution methods to a new level if more comprehensive and accurate cell type information can be included during model building.
